# Supplementary material for: Lexical competition in the flankers task revisited
Source: PLoS One. 2023 Sep 28;18(9):e0285292. doi: 10.1371/journal.pone.0285292 (PMC10538709; doi:10.1371/journal.pone.0285292)
Supplement: S1 Appendix — (DOCX) [file pone.0285292.s001.docx]

**Appendix. Bayesian LMM analyses of the results of Experiment 2.**

|  | Flanker condition | |  |  | Bayesian LMM | | |  |  |  |  |
| --- | --- | --- | --- | --- | --- | --- | --- | --- | --- | --- | --- |
| Error Type | Related | Unrelated | effect |  | *estimate* | *error* | *Lower bound* | *Upper bound* | *^R* | *Bulk ESS* | *Tail ESS* |
| No response | 0.4 % | 0.3 % | -0.1 % |  | -0.20 | 0.44 | -1.07 | 0.65 | 1.00 | 92565 | 50984 |
| Repetition error | 4.9 % | 0.8 % | -**4.1 %** |  | -1.53 | 0.26 | **-2.06** | **-1.04** | 1.00 | 98754 | 52936 |
| Related error | 4.7 % | 5.9 % | 1.2 % |  | 0.25 | 0.15 | -0.03 | 0.54 | 1.00 | 122607 | 51192 |
| Unrelated error | 1.8 % | 2.6 % | 1.2 % |  | 0.40 | 0.22 | -0.02 | 0.83 | 1.00 | 96392 | 50769 |
| Total | 11.8 % | 9.6 % | -**2.2 %** |  | -0.28 | 0.11 | **-0.47** | **-0.07** | 1.00 | 101034 | 51290 |

**Table A1**. Bayesian LMM analysis of effects of flanker relatedness for the different error categories in the word flanker condition.

|  | Flanker condition | |  |  | Bayesian LMM | | |  |  |  |  |
| --- | --- | --- | --- | --- | --- | --- | --- | --- | --- | --- | --- |
| Error Type | Related | Unrelated | effect |  | *estimate* | *error* | *Lower bound* | *Upper bound* | *^R* | *Bulk ESS* | *Tail ESS* |
| No response | 0.1 % | 0.1 % | -0.0 % |  | -0.22 | 0.70 | -1.60 | 1.15 | 1.00 | 137273 | 49243 |
| Repetition error | 3.7 % | 0.1 % | **-3.6 %** |  | -2.84 | 0.46 | **-3.79** | **-1.99** | 1.00 | 73261 | 50506 |
| Related error | 2.5 % | 8.3 % | **5.8 %** |  | 1.32 | 0.19 | **0.94** | **1.70** | 1.00 | 83442 | 56810 |
| Unrelated error | 3.3 % | 6.0 % | **2.7 %** |  | 0.62 | 0.19 | **0.26** | **0.99** | 1.00 | 70647 | 53553 |
| Total | 9.6 % | 14.5 % | **4.9 %** |  | 0.53 | 0.13 | **0.28** | **0.78** | 1.00 | 70664 | 53373 |

**Table A2**. Bayesian LMM analysis of flanker relatedness for the different error categories in the bigram flanker condition.
