# Supplementary material for: Lexical competition in the flankers task revisited
Source: PLoS One. 2023 Sep 28;18(9):e0285292. doi: 10.1371/journal.pone.0285292 (PMC10538709; doi:10.1371/journal.pone.0285292)
Supplement: Attachment — Submitted filename: Response to Reviewers.pdf [file pone.0285292.s002.pdf]

Dear Dr Tsang,

We thank you and the reviewers for the time and comments, and for inviting us to resubmit a revision of our paper. We were pleased to see that the reviewers recommend publication barring minor revisions. The comments of the reviewers mainly ask about elaboration and additional analyses. As such, our point-by-point answers to these comments can be found below. We hope that these responses are deemed appropriate. All changes are indicated in red colored text in the revision.

Kind regards,

Aaron Vandendaele & Dr Jonathan Grainger

Reviewer #1:

Review of ms “Lexical competition in the flankers task revisited”

This is a nice manuscript using the reading version of the flankers task (Dare & Shillcock, 2013) that examined lexical competition. The topic is interesting, the experiments have been competently conducted and analyzed, and the manuscript is easy to read. Thus, this is a strong candidate for publication. I have several suggestions from improvement:

-It would be better to start the Introduction with a more general paragraph indicating the importance of the topic. The beginning of “In the present study we provide a further examination of the impact of orthographic relatedness across adjacent words within a sequence of words presented simultaneously and aligned horizontally” can be put in context. In this same paragraph, the parenthetical note “e.g., “The slight blue of the lights …” => “The slight blur of the lights …”, where the target word the word “blur” and the preview the word “blue”” seems to miss a couple of verbs (e.g. “where the target word is the word “blur” and the preview is the word “blue”).

Response: We have added a new introductory paragraph in order to provide a better context for the present work, and we have corrected the parenthetical note – thanks for pointing out

this error. Moreover, when re-writing this section, we discovered that we had not done a good job in describing the different paradigms, so what was the initial paragraph of the manuscript has been re-written, and the part concerning parafoveal-previews has been simplified and moved to the new introductory paragraph.

-Related to page 5, there is evidence when manipulating a word's N in lexical decision vs. sentence reading: the same materials produce facilitation in lexical decision and inhibition in reading (including gaze durations, see Pollatsek et al., 1999), so some rewriting may be in order.

Response: We now cite Pollatsek et al. in the new introductory paragraph, and we now refer to this work when discussing possible differences between lexical decision and sentence reading (see new footnote 1).

-On page 9, I would update the reference to lme4. Also, I believe it's more important to indicate the version of R (i.e., the core) than the version of RStudio.

Response: we now cite the correct version of R. We cite the lme4 package as it is referred to in the repository (i.e., https://cran.r-project.org/web/packages/lme4/citation.html). We could not find a more recent version for the reference, but we now mention the version of lme4 which we used for the analyses.

-On page 11, the "1 undefined" was actually "undefined" or rather "didn't say"?

Response: This refers to the option 'other/undefined' given to participants when asked to indicate their gender. We have better specified this in the revision.

-footnote 1: "the posterior distribution did not include 0". I believe the authors wanted to say "the 95% credible interval of the posterior distribution did not include 0".

Response: That is correct, we have now corrected this in the footnote.

-In the General Discussion, the authors may want to spell out in more detail the implications of their study for the models of reading and word recognition. The manuscript ends a bit in an abrupt manner now.

Response: We have added a new paragraph at the end of the General Discussion that summarizes the findings and points to their implications for models of word recognition and reading.

-Figure 2 does not reflect the actual timing of the flankers in the current experiments, so it needs to be slightly modified.

Response: We apologize for this error. The figure has now been modified to display the correct timing.

-Table A1/A2. The values of R need to be with two decimal points (e.g., 1.30 would reflect a bad fit among the chains and this would still be "1").

Response: The Rhat (R^) convergence statistic is now reported up to two decimal points.

So I am quite positive, most of the required work is on some careful rewriting and proofreading—I may have missed a few others.

Response: Many thanks for your positive evaluation of this work. We have done the careful proofreading that you requested, and we hope that all is in order now.

Reviewer #2: This paper reports two online experiments investigating task demands on the flanker effect during word identification. Participants made lexical decisions (Experiment 1) or identified target words (Experiment 2) in the presence of flanker words/bigrams. The results showed that related flanker words facilitated lexical decisions, replicating previous research. Related flanker words, however, were associated with increased errors in the perceptual identification task, whereas related bigrams yielded

facilitation. I have a few queries that I would like the authors to address before recommending this paper for publication.

- P. 5: I think it would be useful to elaborate a bit why it is argued that "the decision to move the eyes to the next word in a sequence" does not require unique word identification. The authors cite the E-Z Reader model to support this but, although that model assumes that the trigger to begin programming a saccade to the next word is the completion of a preliminary stage of word identification, it doesn't seem accurate to imply that unique word identification does not occur in this model.

Response: We have now clarified this point. It is the fact that in EZ-Reader it is the familiarity check and not word identification that triggers the decision to move the eyes to the next work that is crucial for our arguments. We have clarified this point.

- P. 8: Please clarify if the 2025 observations per condition were based on trials before or after the data exclusion procedures reported in the following section.

Response: This number of observations per condition is before data exclusion procedures. We did not apply an adaptive testing procedure to guarantee enough observations per condition. Rather, we based ourselves on data from previous studies which used a similar experiment setup. We estimated a margin of 20%, meaning 2000 observations was our pre-set goal. After all exclusion procedures, we ended up with 1601 observations in the orthographically related flanker condition and 1474 in the control flanker condition. We now mention this in the paper.

- P. 9: It is stated that the LMM models only included subject and item random intercepts "if the model structure allowed for it". Please elaborate on the model selection procedure. I would be surprised if the models failed to converge with random intercepts. Perhaps this is a typo – did you mean random slopes here?

Response: This was indeed a typo. To clarify this, we now specify the model selection procedure. Next to that, the final model for each analysis has been written out.

- The Experiment 2 data is analysed separately for the two sub experiments. Given that the targets were exactly the same in the two conditions, I think an analysis that combines the data and includes flanker type as a factor (and tests interactions with flanker relatedness) would be preferable.

Response: We now present the analysis of Experiment 2 as recommended by this reviewer. We obtain the same pattern of results, but with the addition of a significant interaction effect between type of flanker and flanker relatedness. This significant interaction allows us to examine, as we did before, the effects of flanker relatedness separately for the word and bigram flankers. We have also included a new figure (Figure 3) that provides an overview of these results and that highlights the significant interaction.
